# 30 Years Since the Proposal of Exon Skipping Therapy for Duchenne Muscular Dystrophy and the Future of Pseudoexon Skipping

**DOI:** 10.3390/ijms26031303

**Published:** 2025-02-03

**Authors:** Masafumi Matsuo

**Affiliations:** Graduate School of Science, Technology and Innovation, Kobe University, Kobe 657-8501, Japan; matsuo@kobe-u.ac.jp; Tel.: +81-78-803-6495

**Keywords:** Duchenne muscular dystrophy, exon-skipping therapy, antisense oligonucleotides, pseudoexon skipping

## Abstract

Thirty years ago, in 1995, I proposed a fundamental treatment for Duchenne Muscular Dystrophy (DMD) using antisense oligonucleotides (ASOs) to induce exon skipping and restore dystrophin expression. DMD is a progressive and fatal muscular dystrophy, and the establishment of an effective therapy has been a pressing demand among patients worldwide. Exon-skipping therapy utilizing ASOs has garnered significant attention as one of the most promising treatments for DMD, stimulating global research and development efforts in ASO technology. Two decades later, in 2016, one ASO was conditionally approved by the U.S. FDA as the first DMD treatment. This review summarizes the current status and challenges of ASO-based exon-skipping therapies for DMD and explores the prospects of pseudoexon skipping using ASOs, which holds the potential for achieving a complete cure for DMD.

## 1. Introduction

Duchenne Muscular Dystrophy (DMD) is a progressive muscular dystrophy caused by mutations in the *DMD* gene that result in the absence of dystrophin. The disease manifests in childhood with symptoms of muscle weakness and progresses to severe complications such as heart failure, with most patients succumbing before the age of 30. This fatal disease affects approximately one in 5000 male births worldwide, irrespective of race, and the establishment of an effective treatment has been a pressing need among patients globally [1]. In 1995, I proposed a fundamental therapeutic strategy for DMD, utilizing antisense oligonucleotides (ASOs) to induce exon skipping and restore dystrophin expression. Two decades later, in 2016, the first ASO received conditional approval from the U.S. FDA as a treatment for DMD. Subsequently, three additional ASOs were approved by the FDA for the treatment of DMD, leading to the widespread recognition of exon skipping as a therapeutic approach for this disease. This article summarizes the current status and challenges of ASO-based exon-skipping therapies for DMD. Additionally, certain disease-causing variants in the *DMD* gene result in the formation of pseudoexons. The successful skipping of these pseudoexons could potentially lead to a complete cure for DMD. Thus, I also review pseudoexon-related disease-causing variants and explore the potential of ASOs expected to achieve complete disease cure.

## 2. Exon-Skipping Therapy Using ASOs for DMD

### 2.1. Proposal of Exon-Skipping Therapy Using ASOs

The groundbreaking therapeutic strategy of exon skipping using ASOs for DMD was conceived based on the molecular pathology analysis of a single DMD patient [2]. When the DMD gene was cloned as the causative gene for DMD in 1987 [3], gene replacement therapy involving the introduction of the *DMD* gene into patients was considered a promising treatment approach [4]. However, due to the enormous size of the *DMD* gene and challenges related to its delivery into muscle cells, practical implementation was deemed a distant goal. A breakthrough came in 1990 when our team identified a 54-base deletion mutation within exon 19 of the *DMD* gene [5]. This mutation, the smallest mutation identified in the *DMD* gene at the time, was named “Dystrophin Kobe”. Unexpectedly, despite being a small in-frame mutation, it caused exon 19 skipping during splicing, a surprising outcome [6].

Splicing involves the precise removal of introns, and the recognition of splice sites is tightly regulated. For this reason, consensus sequences are conserved upstream of the 5′ end of the exon as the branch point and splice acceptor site and downstream of the 3′ end of the exon as the splice donor site. Furthermore, splicing enhancer or splicing silencer sequences are present within exons or introns, ensuring the functionality of these consensus sequences. In the case of Dystrophin Kobe, the deletion of a splicing enhancer sequence led to exon 19 skipping, as demonstrated through splicing assays using mini-genes [7]. At the same time, it was revealed that an ASO complementary to the deleted splicing enhancer sequence could inhibit splicing [7].

**Figure 1:**
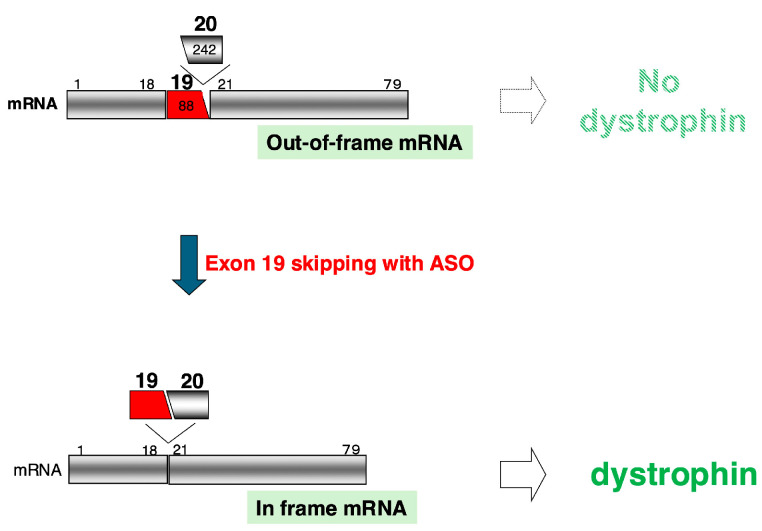
Example of DMD therapy inducing exon skipping with an ASO.

Based on these findings, I proposed in 1995 that “inducing exon skipping during splicing of the *DMD* gene using ASO could correct the reading frame and restore dystrophin production”, offering a fundamental treatment for DMD [7]. This approach aims to convert the out-of-frame *DMD* mRNA in patients to an in-frame transcript, enabling the production of a truncated yet functional dystrophin protein (Figure 1). Although derived from the analysis of a single patient, this concept represented a novel therapeutic strategy to restore the function of mutated genes.

The mechanism of DMD treatment through exon skipping induced by an ASO is illustrated in a schematic diagram, using the case of the world’s first DMD patient treated with this method as an example. In a patient with a 242-base deletion of exon 20 (white box), the mRNA reading frame is shifted, resulting in an out-of-frame mRNA and the absence of dystrophin production (upper). By inducing the skipping of the 88-base exon 19 (red box) using an ASO, both exons 19 and 20 are removed from the mRNA. This results in a total deletion of 330 bases in the mRNA, restoring the in-frame reading frame and enabling the production of dystrophin (lower). The black boxes represent exons, the numbers above the boxes indicate exon numbers, and the numbers inside the boxes show the base count encoded within each exon.

The efficacy of this groundbreaking therapy was demonstrated by results showing that the synthesized ASO successfully induced exon 19 skipping [8] and restored dystrophin expression in cultured cells from a patient with an exon 20 deletion [9]. In 2003, ASO therapy was administered to a DMD patient for the first time worldwide, and dystrophin expression resulting from exon 19 skipping was confirmed [10]. This success attracted significant attention to ASO-based exon skipping as one of the most promising therapeutic approaches for DMD and greatly accelerated ASO development [2,11]. Marking the 30th anniversary of the therapeutic proposal, a reevaluation of the world’s first patient treated with this therapy was conducted 20 years later. Remarkably, it was found that the patient’s cardiac function had been exceptionally well preserved [12].

### 2.2. ASOs Approved as Therapeutics for DMD

The ASOs used for exon-skipping therapy in DMD are specific to the type of exon deletion mutations that patients have. Consequently, the development initially focused on ASOs that induce the skipping of exon 51, as this targets the largest patient population [13,14]. In 2007, Drisapersen (PRO0051), composed of 2′-O-methyl-phosphorothioate RNA (2′-O Me), was demonstrated to induce dystrophin expression in cultured cells from affected patients [15], leading to the initiation of clinical trials. Unfortunately, Drisapersen’s development was halted due to insufficient efficacy and adverse effects [16].

On the other hand, ASOs utilizing morpholino nucleotides to induce exon 51 skipping were developed. Following clinical trials, this ASO was conditionally approved by the U.S. FDA in 2016 as Eteplirsen [17]. This marked a significant milestone in the treatment of DMD, offering new hope to patients worldwide who previously had no available therapies [18]. Subsequently, development progressed for ASOs targeting other exons with large patient populations, particularly exon 53 and exon 45 [19,20]. Currently, in addition to Eteplirsen, three other ASOs have received conditional approval from the U.S. FDA: Golodirsen and Viltolarsen, which induce exon 53 skipping [21,22], and Casimersen, which induces exon 45 skipping [23] (Table 1). However, the patients eligible for treatment with these four ASOs remain limited, highlighting the need for the development of additional ASOs [24].

## 3. Development of ASO Therapeutics Inspired by Exon-Skipping Therapy for DMD

### 3.1. ASOs for Suppressing Exon Skipping

The use of ASOs to induce exon skipping demonstrates a method by which ASOs can artificially modulate splicing, leading to numerous advances in ASO-based therapies [25,26]. Contrary to DMD treatment, an ASO has also been developed to suppress exon skipping. Spinal muscular atrophy (SMA) is a motor neuron disease caused by mutations in the *SMN1* gene. In SMA, inhibiting exon 7 skipping in the structurally similar *SMN2* gene can restore SMN protein expression and serve as a treatment [27]. Accordingly, an ASO that inhibits the function of intronic splicing silencer sequence, promoting exon 7 inclusion, was developed as a therapeutic for SMA and was approved by the FDA as Nusinersen in 2016 [28].

### 3.2. Expansion of Exon-Skipping Therapy

Exon skipping modifies the nucleotide sequence of mRNA, and beyond restoring gene function in DMD, it has also been applied to disrupt or modify gene function. In a 2021 review, I reported that exon skipping had been investigated as a therapeutic strategy for more than 25 genes [2]. Recent developments include ASOs designed to skip exons with nonsense mutations in the *PRPF31* gene for retinitis pigmentosa [29] and the *CFTR* gene for cystic fibrosis, aimed at restoring gene function [30]. Similarly, an ASO targeting exon 12 of the *HTT* gene has been developed to skip the exon, eliminating the caspase-6 cleavage site from the resulting HTT protein, thereby preventing degradation by caspase-6. This ASO is currently being developed as a treatment for Huntington’s disease [31].

### 3.3. New Trends in Exon-Skipping Applications

**Figure 2:**
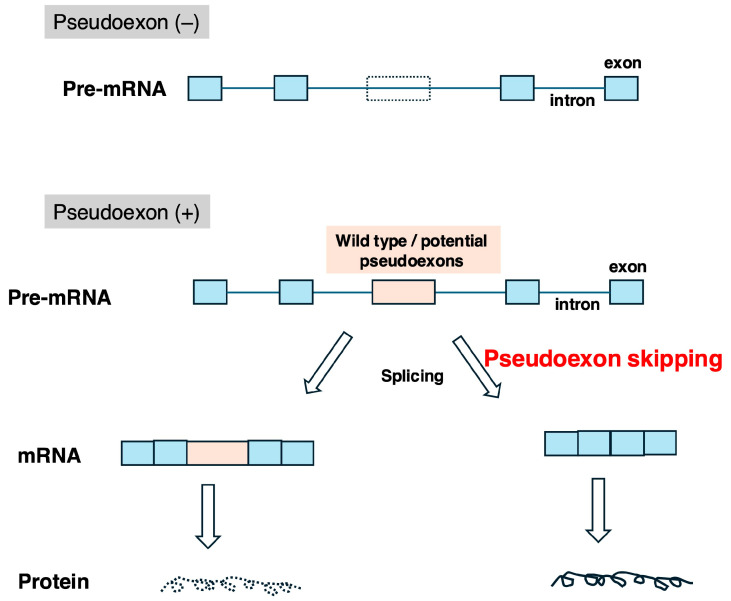
Pseudoexons and their skipping. In normal conditions, pre-mRNA is composed only of authentic exons, producing normal mRNA (pseudoexon (–)). However, due to certain genetic disease-causing variants, a pseudoexon (either a wild pseudoexon or a potential pseudoexon) can form within an intronic sequence (pseudoexon (+)). If this pseudoexon is incorporated into mRNA during splicing, protein synthesis is disrupted (left). Using an ASO to induce the skipping of the pseudoexon allows the production of completely normal mRNA and proteins (right). Blue rectangles represent normal exons, while orange rectangles indicate pseudoexons.

Certain genetic disease-causing variants result in the formation of novel exons (pseudoexons). In such cases, inducing pseudoexon skipping can enable the production of completely normal mRNA, potentially leading to a complete cure of the disease (Figure 2). Clinical trials using ASOs to induce pseudoexon skipping are underway for Leber’s disease [32]. Additionally, ASOs targeting cryptic exon skipping are being developed for ALS [33]. One noteworthy application of pseudoexon skipping is the development of ASOs tailored for individual patients. For instance, in Batten disease, an ASO successfully induced the skipping of a pseudoexon in the *CLN7* gene, enabling the production of functional mRNA and restoring gene function [34]. This success has spurred global efforts to develop “N-of-1” ASO therapies [35]. Our team also identified an ASO capable of inducing pseudoexon skipping in the *WDR45* gene for a case of beta-propeller protein-associated neurodegeneration (BPAN) [36]. Thus, ASOs that induce exon skipping are opening new therapeutic horizons for previously untreatable diseases.

## 4. Challenges and Improvements for ASO Drugs in DMD Treatment

### 4.1. Challenges of Approved ASO Drugs

Eteplirsen, the first FDA-approved drug for DMD, received exceptional approval despite concerns such as the low level of dystrophin expression in skeletal muscle (<1% of normal), which is a key indicator of therapeutic efficacy, and the small number of patients enrolled in clinical trials [17]. Consequently, although approved in the United States, it was rejected in Europe [37].

Recently, cautionary reports regarding conditionally approved ASO drugs have begun to emerge. First, questions have been raised about whether the data submitted for FDA approval were rigorously evaluated [38]. Second, additional post-approval investigations have revealed efficacy-related issues. Viltolarsen, which induces exon 51 skipping, was considered a highly promising therapeutic agent [20]. However, the company recently reported that confirmatory tests showed no significant difference in time to functional improvement compared to the placebo group (https://www.nspharma.com/pdfs/%5BNSP%5D_Press_Release_RACER53_Study_Results.pdf) (accessed on 30 November 2024). The third issue involves side effects. Some approved ASO drugs exhibit adverse effects [39], including nephrotoxicity, which the FDA has flagged as a warning. Given that DMD therapies require lifelong administration, the potential for cumulative toxicity poses a significant concern. Additionally, the development of Vesleteplirsen, which induces exon 51 skipping, was recently halted following reports of hypomagnesemia during clinical trials (https://www.sarepta.com/community-letter-update-srp-5051-program) (accessed on 30 November 2024).

### 4.2. Improvements in ASOs for DMD Treatment

Even for approved ASOs, improvements are necessary due to their low efficacy, and various methods to improve ASOs and related technologies are under investigation. Administered ASOs circulate in the bloodstream and are distributed throughout the body. During this process, the efficiency of ASO delivery to muscle cells becomes a critical factor in their effectiveness. To enhance the delivery efficiency to tissues, ASOs conjugated with ursodeoxycholic acid have been developed to increase lipid affinity, improve membrane permeability, and allow absorption from the gastrointestinal tract [40]. Furthermore, even if ASOs enter muscle cells, they may be sequestered into endosomes and directed toward degradation pathways, reducing their efficacy [41]. To address this, chemical modifications to prevent endosomal sequestration are being investigated [42].

Within the nucleus, ASOs bind to their target pre-mRNA and induce exon skipping. To improve the efficiency of exon skipping, an ASO targeting two sequences simultaneously within an exon was developed to induce the skipping of exon 44, showing promising results [43]. Additionally, ASOs composed of 5′-methylcytosine and locked nucleic acid (LNA) were developed to enhance exon 51 skipping efficiency, resulting in significantly increased dystrophin expression in model mice [44]. However, these advances in research have led to unexpected clinical challenges [45]. Specifically, in Europe, the increase in clinical trials for ASOs inducing exon 51 skipping has sparked debates over which ASO to prioritize for patient treatment. Although this may seem like a fortunate dilemma, the limited number of eligible patients necessitates more efficient clinical trials, representing a challenge for the future.

ASOs with strong exon-skipping effects are highly desirable. For instance, an ASO composed of LNA-2′O Me and LNA-FRANA was developed to induce exon 53 skipping with high efficiency [46]. However, despite demonstrating strong exon-skipping effects in skeletal and cardiac muscles, dystrophin expression was lower than expected. This discrepancy between mRNA and protein levels raised concerns about ASO artifacts, such as the preferential amplification of smaller fragments during PCR or the impact of LNAs with high complementary binding on cDNA synthesis and PCR amplification [46]. Similar issues have been observed with ASOs using tricyclo-DNA, which showed exaggerated exon-skipping effects [47]. Therefore, the development of ASOs requires careful evaluation to avoid overestimating their exon-skipping capabilities.

When ASOs induce the production of the target mRNA, it must be efficiently translated into protein. In DMD, miR-146a binds to the 3′ untranslated region of *DMD* mRNA and suppresses its translation. Knocking out miR-146a in mice increased dystrophin expression, suggesting that miR-146a inhibition could enhance dystrophin levels even with limited mRNA [48].

Increasing the number of muscle cells, which are diminished in DMD, is another critical factor for enhancing ASO efficacy. Various myostatin inhibition strategies have been developed, but no myostatin inhibitors have yet been approved [49,50]. We identified a novel isoform of myostatin, myostatin-b, which has inhibitory effects on myostatin activity [51]. Currently, we are developing splicing-switch ASOs to promote myostatin-b expression, providing a dual inhibition of myostatin. This approach is expected to represent a novel and powerful strategy for muscle cell proliferation and hypertrophy.

Cardiac dysfunction is a major determinant of life expectancy in DMD, making its management a crucial aspect of treatment [52,53]. However, all FDA-approved ASOs for DMD are composed of morpholino nucleotides, which have low delivery efficiency to cardiac muscle. To address this issue, ASOs with improved cardiac delivery are being developed [54]. Interestingly, a simple ASO has shown potential for cardiac delivery. For example, the subcutaneous injection of an ENA-based ASO, Renadirsen, in cynomolgus monkeys successfully delivered the ASO to cardiac tissue [55].

These findings highlight the need for optimization across multiple steps to enhance the efficacy of ASOs in DMD treatment.

## 5. Therapeutic Strategies for Inducing Skipping of Pseudoexons in the *DMD* Gene

Pseudoexons are broadly categorized into two types: wild-type pseudoexons and potential pseudoexons (Figure 2) [56]. The former refers to sequences that originally have exon-like structures but are incorporated into mRNA due to disease-causing variants in splicing enhancer or silencer sequences. The latter are incorporated into mRNA as a result of variants forming splicing consensus sequences. Reports of genetic disease-causing variants leading to pseudoexon formation have been increasing with advances in gene analysis technologies [56,57,58]. In the DMD gene, disease-causing variants can result in the insertion of pseudoexons into mRNA, causing dystrophinopathies [59]. Additionally, there are reports of pseudoexon insertions in the DMD gene whose pathological significance remains unclear [60]. If the skipping of disease-causing pseudoexons in DMD is successfully induced, completely normal mRNA will be produced, potentially leading to a complete cure of DMD (Figure 2). Therefore, the development of ASOs to induce pseudoexon skipping holds therapeutic potential for dystrophinopathies [57,61]. However, as previously mentioned, ASO drug development for DMD has prioritized mutations affecting larger patient populations, leaving the development of ASOs targeting pseudoexon skipping relatively underexplored. Given the severity of DMD, it is reasonable to consider pseudoexon-skipping ASOs even for “N-of-1” treatments [62]. To facilitate further development, this review summarizes reported mutations in the *DMD* gene that result in pseudoexon formation.

### 5.1. Identified Pseudoexons in the DMD Gene

**Figure 3:**
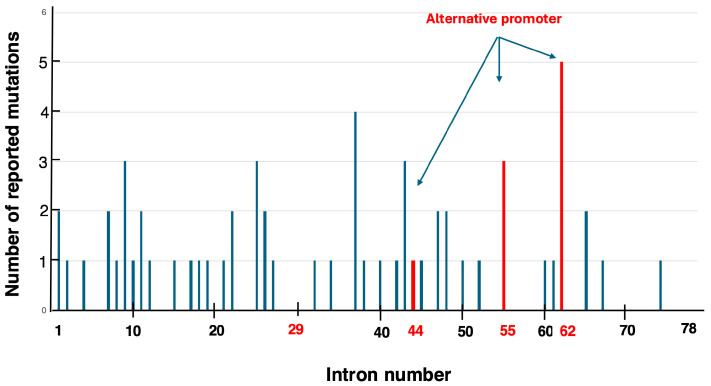
Distribution of pseudoexon-forming genetic variants across introns. A bar graph illustrates the number of pseudoexon-forming genetic variants identified in each intron of the *DMD* gene, spanning introns 1 to 78. The x-axis represents the intron numbers, while the y-axis indicates the number of reported cases. Red bars denote introns harboring alternative promoters of the *DMD* gene. Notably, intron 62 has the highest number of reported disease-causing variants, with five identified variants.

A total of 61 single-nucleotide disease-causing variants leading to pseudoexon formation in the DMD gene have been reported, based on the author’s review of the literature [58,63,64,65,66,67] (Supplementary Table S1). The distribution of these variants within the DMD gene spans introns 1 to 74 (Figure 3). While most introns contained a single disease-causing variant, introns 9, 25, 43, and 55 harbored three disease-causing variants each, and intron 62 contained five disease-causing variants, suggesting disease-causing variant clustering within specific introns. Intron 62 is home to the Dp71 promoter, one of the alternative promoters of the DMD gene, implying a potential relationship between disease-causing variant clustering and alternative promoters. Further investigation into introns 29, 44, and 55, which also contain alternative promoters, revealed three disease-causing variants in intron 55. Thus, two out of the four introns with alternative promoters showed disease-causing variant clustering, hinting at a connection between alternative promoters and the occurrence of pseudoexon-forming disease-causing variants. Future studies are expected to elucidate this relationship.

On the other hand, the high frequency of reported disease-causing variants in certain introns was thought to be related to the size of the introns. The sizes of introns 9, 25, 43, 55, and 62, which have a higher number of reported disease-causing variants, are 52.7 kb, 8.6 kb, 70.4 kb, 120.1 kb, and 62.5 kb, respectively. Among these, intron 55 is notably large, exceeding 100 kb. However, intron 44 in the DMD gene is 248.4 kb in size, making intron 55 the fourth largest intron, following introns 44, 1, and 2. Thus, no clear correlation was observed between intron size and the number of pseudoexon-forming mutation variants.

The frequent occurrence of pseudoexon-forming variants within specific introns suggests the presence of nucleotides that are particularly prone to genetic alteration. Indeed, nonsense mutations caused by single-base substitutions in the *DMD* gene are often attributed to nucleotide changes at CpG dinucleotide sites [68]. Similarly, A>G substitutions have been frequently reported as variants leading to pseudoexon formation in the *DMD* gene. This implies that, like nonsense mutations, CpG dinucleotides may serve as a background factor for base substitutions that result in pseudoexon formation.

### 5.2. Candidate Variants for Pseudoexon-Skipping Therapy

When considering pseudoexon-skipping therapy for the potential complete cure of DMD, it is desirable that a single ASO can treat as many patients as possible. To evaluate this, the number of reports for each pseudoexon-forming variant in the DMD gene was examined (https://www.dmd.nl/) (accessed on 30 November 2024). Most disease-causing variants were reported only once. Surprisingly, among the five variants in intron 62, three were reported only once, while two variants, c.9225–647A>G (−647A>G) and c.9225–285A>G (−285A>G), were reported eight and nine times, respectively. From the perspective of the practical implementation of pseudoexon-skipping therapy, these two highly frequent variants are considered the primary candidates for ASO-based treatment in the future.

**Figure 4:**
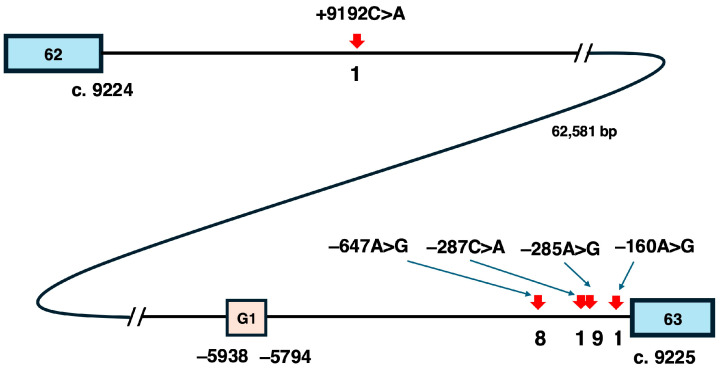
Intron 62 of the *DMD* gene and pseudoexon-forming variants. The approximate locations of five pseudoexon-forming variants within intron 62 and the position of exon G1 are illustrated. Intron 62 is composed of 62,581 bases, and four out of the five variants are located downstream of exon G1, concentrated within 1000 bases upstream of exon 63. The −287C>A and −285A>G variants differ by only two bases in their positions. The numbers below the five variants indicate the frequency of reports. The blue squares represent exons, with the numbers inside denoting the exon numbers. The numbers below these exons correspond to the cDNA base position located at the 3′ end for exon 62 and the 5′ end for exon 63. The orange square indicates the G1 promoter/exon 1 region, while the line represents intron 62.

The positional relationships of these five variants within intron 62, which spans 62,581 bases, were then investigated (Figure 4). Among the five variants, c.9224+9192C>A is located furthest upstream, followed by −647A>G, c.9225–287C>A (−287C>A), −285A>G, and c.9225–160A>G. Notably, the four downstream variants are concentrated within 1000 bases between the G-promoter/exon 1 region and exon 63. Of these, −647A>G and −285A>G, reported eight and nine times respectively, are separated by only 362 bases. In contrast, −287C>A is located just two bases upstream of −285A>G and is reported only once, despite being situated between the two frequently reported variants. This suggests that the occurrence of these variants is not simply influenced by genomic structure but may be associated with specific factors at the affected base.

On the other hand, repetitive *Alu* sequences in the human genome are often reported to undergo exonization [69]. Similarly, the exonization of *Alu* sequences in the *DMD* gene has been reported [70]. Notably, pseudoexons resulting from Alu exonization have been found to be identical in the 6-pyruvoyl-tetrahydropterin synthase (PTS) gene and the coagulation factor 8 (F8) gene [71]. This suggests that a single ASO that induces pseudoexon skipping could potentially treat multiple diseases. Therefore, if the *DMD* pseudoexon were formed through *Alu* exonization, it might share commonalities with pseudoexons in other diseases. To investigate this possibility, the genomic sequences containing the two highly frequent pseudoexon-forming variants were analyzed using Repeat Masker (https://www.repeatmasker.org/) (accessed on 30 November 2024). Contrary to expectations, no *Alu* or other repetitive sequences were found within the regions examined. At present, it is believed that *DMD* pseudoexons do not share common features with pseudoexons in other genes.

**Figure 5:**
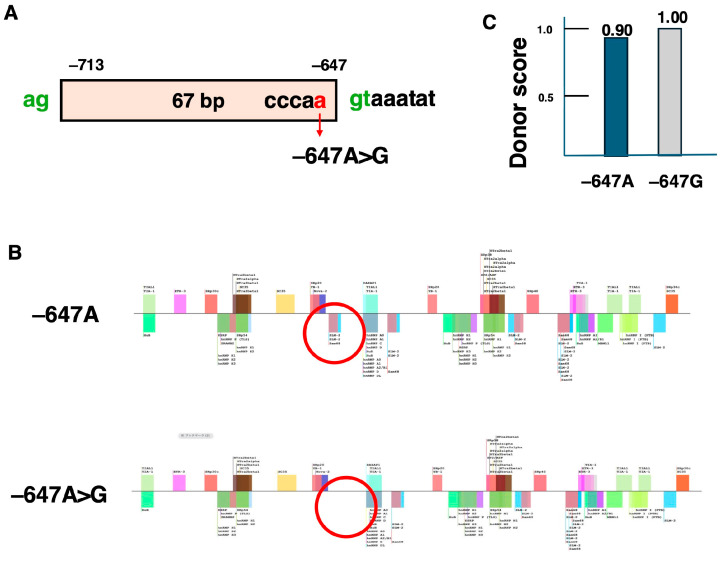
Pseudoexon generated by c.9225–647A>G. The structure of the pseudoexon generated by the c.9225–647A>G (−647A>G) variant in intron 62 is schematically shown (**A**). This −647A>G mutation caused the 67 bases between positions −713 and −647, flanked by ag and gt, to become a pseudoexon. The −647A>G variant was located at the 3′ end of this pseudoexon. The splicing regulatory protein binding sites affected by this variant were analyzed using SpliceAid2 (**B**). The bars above and below the line represent binding sites for splicing-enhancing and splicing-silencing nuclear proteins, respectively. In the normal (−647A) sequence, binding sites for splicing-suppressing proteins (circled in red) were present. However, this binding site disappeared in the −647A>G sequence, indicating that the variant resulted in the loss of the splicing-silencer sequence. Furthermore, the donor score for the 5′ splice donor site was calculated (**C**). In the normal −647A sequence, the donor score was 0.90, while it increased to 1.00 in the −647A>G sequence.

To induce the skipping of pseudoexons, it is essential to design ASOs targeting the key sequences that regulate splicing. The activation mechanisms of the two aforementioned pseudoexons were investigated. First, in the case of −647A>G, 67 bases between the ag and gt splicing consensus sequences from position −713 to −647 were incorporated as a pseudoexon (Figure 5A). The intronic sequence, which originally contained the splicing consensus sequences ag and gt, underwent a variant from A to G immediately upstream of the gt site, leading to its incorporation into mRNA as a pseudoexon (Figure 5A). The change in splicing regulatory protein binding motifs caused by this variant was analyzed using SpliceAid2 (http://193.206.120.249/splicing_tissue.html) (accessed on 15 October 2024). The analysis revealed that a binding site for a splicing repressor protein, which existed at −647A, was lost in −647A>G (Figure 5B). This loss of a splicing repressor motif caused by the variant is thought to be the underlying reason for the pseudoexon inclusion. Furthermore, to evaluate the regulatory elements involved in splicing, the donor score was calculated using the Splice Site Prediction by Neural Network tool (https://www.fruitfly.org/seq_tools/splice.html) (accessed on 15 October 2024). In the wild-type sequence (−647A), the donor score was 0.90, but the score increased to 1.00 following the −647A>G substitution (Figure 5C). This indicates that the −647A>G mutation resulted in the formation of a highly active splice donor site. In summary, the combination of the loss of the splicing repressor motif and the increase in donor score due to the −647A>G variant likely facilitated the inclusion of the pseudoexon.

**Figure 6:**
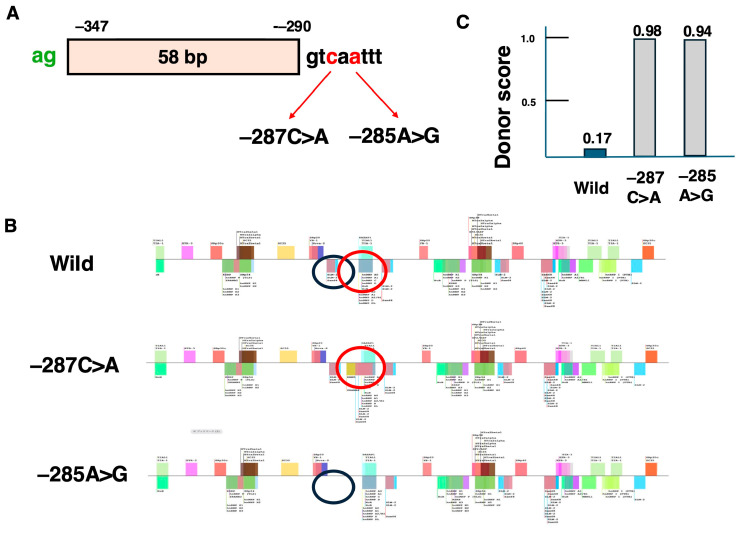
Pseudoexon generated by c.9225–285A>G and c.9225–287C>A variants. The structure of the pseudoexon generated by c.9225–285A>G (−285A>G) and c.9225–287C>A (−287C>A) variants in intron 62 are schematically shown (**A**). For both variants, the 58 bases betweenpositions −347 and −290, flanked by ag and gt, form a pseudoexon. Changes in the splicing regulatory sequences caused by the −287C>A and −285A>G variants were analyzed using SpliceAid2 and are shown in (**B**). For −287C>A, a binding site for splicing-suppressing proteins (indicated by a red circle) was newly added, forming a splicing-silencer sequence. This finding did not align with the observed pseudoexon insertion. Therefore, donor scores were calculated (**C**). The donor score for the wild-type (−287C) was only 0.17, but it significantly increased to 0.98 for −287C>A. Thus, the pseudoexon formation caused by −287C>A was attributed to the increased donor score. For −285A>G, changes in the splicing regulatory sequences were also analyzed (**B**). In the wild-type sequence, binding sites for splicing-suppressing proteins (indicated by a black circle) were present but disappeared in the −285A>G variant. This result indicated that pseudoexon formation was due to the loss of the splicing silencer sequence. Furthermore, the donor score for the −285A>G variant was calculated to be 0.94, much higher than that of the wild-type (**C**). These findings suggest that the pseudoexon formation caused by −285A>G resulted from both the loss of the splicing-silencer sequences and increases in donor scores.

The other frequently reported variant, −285A>G, was analyzed next. Interestingly, both −285A>G and −287C>A variants shared a common pseudoexon formed by 58 bases flanked by ag and gt sequences between positions −347 and −290 in the intron (Figure 6A). Using SpliceAid2, the binding sites for splicing regulatory nuclear proteins affected by these variants were analyzed. (Figure 6B). For the −287C>A variant, a new binding site for splicing repressor nuclear proteins, absent in the wild-type −287C sequence, was added. This resulted in the creation of a splicing silencer sequence, which theoretically suppresses pseudoexon insertion. However, this finding contradicted the observed pseudoexon inclusion. Therefore, the donor score, which reflects the strength of the splice site, was calculated. For the wild-type −287C sequence, the donor score was only 0.17, whereas it significantly increased to 0.98 in −287C>A. (Figure 6C). This suggests that the formation of the pseudoexon by−287C>A was primarily due to the increased donor score.

In contrast, SpliceAid2 analysis of the more frequently reported −285A>G variant revealed the loss of binding sites for splicing repressor proteins, unlike −287C>A (Figure 6B). This loss indicated that pseudoexon formation resulted from the disappearance of the splicing silencer sequence. Additionally, the donor score for −285A>G was 0.94, much higher than that for the wild-type −285A sequence. (Figure 6C). These findings suggested that pseudoexon formation due to the −285A>G variant was driven by both the loss of the splicing silencer sequence and the increased donor score, facilitating splicing.

These results demonstrated that the factors leading to pseudoexon formation vary depending on the variant. Both −647A>G and −285A>G, identified as candidates for pseudoexon-skipping therapy, shared two contributing factors: the loss of splicing silencer sequences and the increase in donor scores, which promoted pseudoexonization. Therefore, it was assumed that there would be minimal differences in the effectiveness of exon skipping induced by ASOs between the two pseudoexons. However, since the pseudoexon formed by the 285A>G variant is identical to that formed by the 287C>A variant, a single ASO might effectively induce the skipping of the pseudoexon in both variants. In such a case, the number of patients eligible for treatment with one ASO could increase to 10. This suggests that developing an ASO to induce the skipping of the pseudoexon formed by the 285A>G variant should be prioritized. 

## 6. Final Remarks

An ASO that modulates splicing was first proposed 30 years ago with the concept of exon-skipping therapy for DMD, which has since laid the groundwork for treatments targeting various diseases. They have also played a pivotal role in pioneering new therapeutic approaches, including N-of-1 treatments. In the case of DMD, where ASOs have led the way, there remains a pressing need to further enhance therapeutic efficacy and high hopes for achieving complete cures through pseudoexon skipping.

Nonetheless, significant challenges remain in the practical application of ASOs. Despite being synthesized from just four types of amidites, regulatory authorities treat each ASO as a completely novel synthetic entity. If evaluations could be streamlined to focus solely on nucleotide sequences, the implementation of ASOs could accelerate substantially, delivering profound benefits to patients. Urgent revision of the evaluation criteria for ASO drugs is greatly anticipated.

## Figures and Tables

**Table 1:** Conditionally approved ASO drugs for DMD therapy by the FDA.

Name	Target Exon	Year of Approval
Eteplirsen	51	2016
Golodrisen	53	2020
Viltolarsen	53	2020
Casimersen	45	2021

The four conditionally approved ASO drugs for DMD therapy by the FDA are shown with their respective target exons for skipping and the year of approval.

## Supplementary Materials

The following supporting information can be downloaded at: www.mdpi.com/article/10.3390/ijms26031303/s1.
