# Supplementary material for: 30 Years Since the Proposal of Exon Skipping Therapy for Duchenne Muscular Dystrophy and the Future of Pseudoexon Skipping
Source: Int J Mol Sci. 2025 Feb 3;26(3):1303. doi: 10.3390/ijms26031303 (PMC11818380; doi:10.3390/ijms26031303)
Supplement: Supplementary file 1 [file ijms-26-01303-s001.zip › ijms-3411003-supplementary.pdf]

Supplementary Table S1. Pseudoexon-Forming Variants in the *DMD* Gene

A total of 61 pseudoexon-forming variants have been reported. These variants are categorized by the introns in which they occur and are listed in a table in ascending order of intron number. Bold numbers within the intron numbers indicate introns in which multiple variants have been reported.

intron	variant	intron	variant
**1**	c.31+36947G>A	**37**	c.5326-215T>G
**1**	c.31+84433C>G	**37**	c.5326–5219T>G
2	c.93+5590T>A	38	c.5448+67A>G
4	c.265-463A>G	40	c.5739+362A>G
**7**	c.650-39498A>G	42	c.6117+3363A>G
**7**	c.650-39575A>C	**43**	c.6290+3076A>G
8	c.832-186 T>G	**43**	c.6290+ 30954C>T
**9**	c.960+1789C>G	**43**	c.6291-13537A>G
**9**	c.961-5925A>C	44	c.6438+47818G>T
**9**	c.961-5831C>T	45	c.6614+3310G>T
10	c.1149+250C>T	**47**	c.6913-4037T>G
**11**	c.1331+17811C > G	**47**	c. 6913-5879A>G
**11**	c.1332-11909C>G	**48**	c.7098+1813G>T (
12	c.1482+323C>G	**48**	c.7099-1450A>G
15	c.1813-733A>G	50	c.7310-19A>G
17	c.2169-12884G>T	52	c.7661-1646C>G
18	c.2292+1024G>T	**55**	c.8217+18052A>G
19	c.2380+3317A > T	**55**	c.8217 + 23338A > G
21	c.2803+3252A > G	**55**	c.8217+32103G>T
**22**	c.2949+909C>T	60	c.9085-15519G>T
**22**	c.2949+964G>A	61	c.9163+2510G>A
**25**	c.3432+2036A>G	**62**	c.9224+9192C>A
**25**	c.3432+2240A>G	**62**	c.9225-647A>G
**25**	c.3432+3731G>T	**62**	c.9225-287C>A
**26**	c.3603+820G>T	**62**	c.9225-285A>G
**26**	c.3603+2053G>C	**62**	c.9225-160A>G

27	c.3787-843C>A	**65**	c.9563+1215A>G
32	c.4518+512T>A	**65**	*c.9564-427T>G*
34	c.4846-1469A>G	67	c.9807+2714C>T
**37**	c.5325+1758A>T	74	c.10554-2996T>G
**37**	c.5325+1759G>T		
